# A Review of Current *In Silico* Methods for Repositioning Drugs and Chemical Compounds

**DOI:** 10.3389/fonc.2021.711225

**Published:** 2021-07-22

**Authors:** Binsheng He, Fangxing Hou, Changjing Ren, Pingping Bing, Xiangzuo Xiao

**Affiliations:** ^1^ Academician Workstation, Changsha Medical University, Changsha, China; ^2^ Queen Mary School, Nanchang University, Jiangxi, China; ^3^ School of Science, Dalian Maritime University, Dalian, China; ^4^ Genies Beijing Co., Ltd., Beijing, China; ^5^ Department of Radiology, The First Affiliated Hospital of Nanchang University, Jiangxi, China

**Keywords:** drug repositioning, anti-tumor drug, gene expression, drug target, gene interaction network

## Abstract

Drug repositioning is a new way of applying the existing therapeutics to new disease indications. Due to the exorbitant cost and high failure rate in developing new drugs, the continued use of existing drugs for treatment, especially anti-tumor drugs, has become a widespread practice. With the assistance of high-throughput sequencing techniques, many efficient methods have been proposed and applied in drug repositioning and individualized tumor treatment. Current computational methods for repositioning drugs and chemical compounds can be divided into four categories: (i) feature-based methods, (ii) matrix decomposition-based methods, (iii) network-based methods, and (iv) reverse transcriptome-based methods. In this article, we comprehensively review the widely used methods in the above four categories. Finally, we summarize the advantages and disadvantages of these methods and indicate future directions for more sensitive computational drug repositioning methods and individualized tumor treatment, which are critical for further experimental validation.

## Introduction

Drug repositioning is a new way of applying existing therapeutics to new disease indications. Compared with traditional new drug development methods, the advantage of drug repositioning is that it can reduce the time and cost of drug development, and the drug composition has been proven to be safe in human body, so phase I clinical trials can be skipped (1, 2).

The failure probability of new drugs in the development process is about 90% (3), which leads to high drug development costs. In addition, repurposed drugs can save most of the cost of early research and significantly reduce the transition from laboratory research to clinical treatment. According to a research report released by Deloitte & Touche in 2016, according to the tracking results of 12 large pharmaceutical companies for 6 years, the return on investment of R&D giants dropped from 10.1% in 2010 to 3.7% in 2016. It was also calculated that the average cost of developing a new drug has increased from less than 1.2 billion US dollars to 1.54 billion US dollars, and it takes 14 years to launch a new drug (4). Nosengo concluded that it currently takes more than 10 years to bring a drug to the market, and the average research cost is between $2 billion to $3 billion. Although the number of approved drugs for development remains the same or decreases over time, the cost of research continues to increase. In contrast, some studies suggest that repositioning a known drug costs an average of $300 million, and it takes about six to seven years (5). New solutions are needed to solve the above-mentioned problems in the development of new drugs, including drug repositioning.

Drug repositioning refers to the matching and identification of existing drugs and new indications, and trying to apply newly discovered drugs to the treatment of diseases other than expected diseases (6). In addition, drug repositioning has promoted the development of cancer research (7). Researchers are committed to finding potential drug molecules that can block the exchange of information between cancer cells, and prevent cancer cells from receiving information that promotes their growth and proliferation. At present, *in silico* and activity-based methods are mainly used to determine the feasibility of drug repositioning. *In silico* methods for drug repositioning are affected by drug-to-disease relationships, or the gene expression response of cell lines after treatment. Combining multiple information levels, the relationship network between target and drug can be identified by means of bioinformatics tools and public databases (8, 9). Due to decades of accumulation of structural information between proteins and pharmacophores, the method has gradually become successful. Compared with *in silico* drug repositioning, computerized drug repositioning has become a promising technology with fast speed and low cost (10).

Since the outbreak of Corona Virus Disease 2019 (COVID-19), it spreads rapidly all over the world. There is an urgent need for effective drugs to treat and alleviate the deterioration of this novel Coronavirus (11, 12). Since the development of a new drug is time-consuming and costly, drug reposition is a feasible way to meet this need (13, 14). The treatment of COVID-19 relied on the experience of clinicians (15, 16). So far, some drugs have been proved effective in relieving and improving the symptoms of novel coronavirus pneumonia (17–22). The drugs against the Middle East respiratory syndrome coronavirus (MERS-CoV) and severe acute respiratory syndrome coronavirus (SARS-CoV), such as Lopinavir/ritonavir, have been proved to inhibit many viruses (22, 23). As a nucleoside drug and RNA polymerase (RdRp) inhibitor, remandsivir can inhibit SARS-CoV-2 RdRp, subgenomic mRNA and subviral genomic RNA to block the synthesis of negative chain RNA, thus inhibiting virus replication and antiviral effect (24–26).

In this review, we present the recent progress on *in silico* methods for repositioning drugs and chemical compounds. In particular, we focus on feature-based methods, matrix decomposition–based methods, network-based methods, and reverse transcriptome–based methods. We review the *in silico* popular methods in the four categories separately.

## Feature-Based Methods

*In silico* methods of drug compounds and repositioning drugs aims to identify the relationship network between target and drug, which is achieved through bioinformatics tools and public databases. Therefore, it needs to ensure high-resolution structural information, including drugs targets, gene expression profiles, or disease/phenotype information, which usually produce high-dimensional feature datasets. For instance, the Cancer Cell Line Encyclopedia study (27) contains more than 50000 features, representing the mRNA expression and mutational status of thousands of genes. However, the number of available features is significantly greater than the number of training samples. The use of high-dimensional features can lead to overfitting of the model, in fact, only a few features play a key role in the final prediction of drug sensitivity.

**Figure 1:**
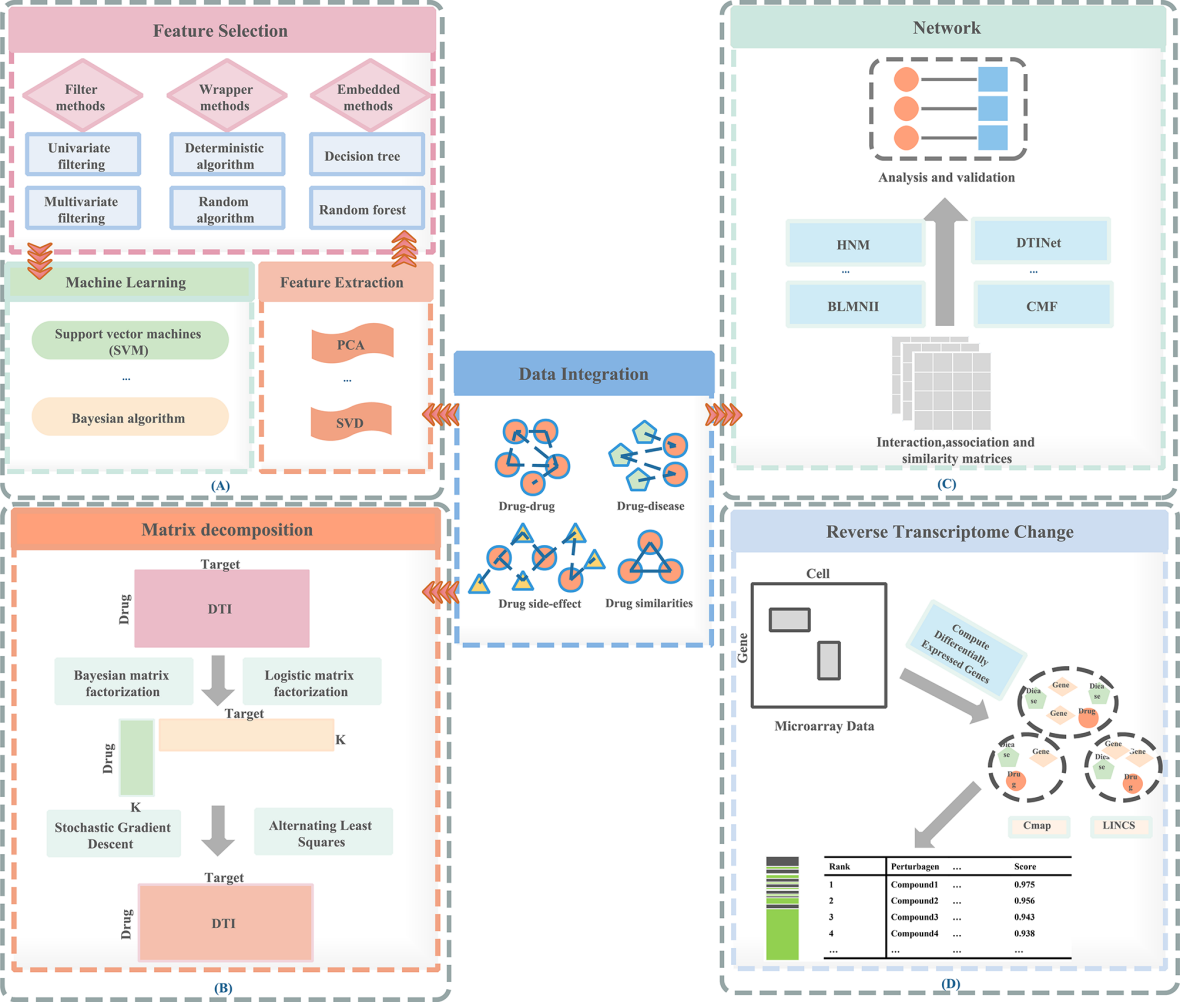
Methods of drug repositioning. **(A)** Feature-based methods, **(B)** matrix decomposition–based methods, **(C)** network-based methods, **(D)** reverse transcriptome change–based methods.

Therefore, a feature-based methods are proposed: (1) can prevent over-fitting and improve model performance; (2) can provide a more cost-effective and faster model; (3) can clearly grasp the basic process of generating data. In **Figure 1A**, we visualize the process of the feature-based method. These are important for understanding the relationships between data in the chemical, clinical domains, and biological fields. Therefore, the research of feature-based drugs sensitivity prediction and individualized treatment methods are very necessary. **Table 1** summarizes the feature-based methods used in a large number of studies.

**Table 1:** Feature-based methods.

Study	Feature extraction	Feature selection			Description	Ref
		Filter methods	Wrapper methods	Embedded methods		
Pearson	√				An effective algorithm for extracting main feature components of data	(28)
Goswami et al.	√				Use principal components analysis (PCA) to identify and remove abnormal samples	(29)
Costello et al.		√			Bayesian multi-task multiple-kernel learning (MKL) method for drug sensitivity prediction and identification	(30)
Robnik-Sikonja et al.		√			Theory and application of ReliefF and RReliefF	(31)
Haider et al.		√			Multivariate ensemble learning regression tree extension ensemble learning method	(32)
De Jay et al.		√			An extended integration method based on mRMR (mRMRe)	(33)
Peng et al.		√	√		A two-stage feature selection algorithm combining mRMR and other feature selection algorithms	(34)
Liu et al.		√			Minimal redundancy and maximal correlation were used to analyze and predict drug interactions	(35)
Pudil et al.			√		Theory and application of floating search method	(36)
Berlow et al.			√		A sensitivity prediction method based on function perturbation data	(37)
Dong et al.			√		Support vector machine recursive feature elimination	(38)
Tikhonov A				√	Ridge regression penalty L-2 norm	(39)
Neto et al.				√	A Bayesian inference method based on ridge regression	(40)
Tibshirani R				√	LASSO penalizes L-1 norm	(41)
Park et al.				√	RRLASSO method of targeted anticancer drugs	(42)
Zou et al.				√	Regularization of elastic networks based on Mixed Penalties of L-1 and L-2 norms	(43)
Das S		√	√		Mixing of filter and wrapper	(44)
Cadenas et al.		√	√		Feature selection based on fuzzy random forest	(45)
I.S. et al.			√		Hybrid genetic algorithms	(46)
Ali et al.		√	√		Hybrid ant colony optimization	(47)
Sarafrazi et al.			√		Hybrid Gravity Search Algorithm	(48)
Sokolov et al.				√	Path-based elastic network regularization	(49)
Bandyopadhyay et al.				√	A feature selection method combining gene expression data with signals and regulatory pathways	(50)
Amadoz et al.			√		Use the activation state of the signal pathway as a feature	(51)

### Feature Extraction and Feature Selection

The purpose of feature extraction is to project features into new low-dimensional feature space. The features after dimension reduction are usually a combination of the original features, with the aim of discovering more representative information through the new feature sets. A common example of feature extraction technique is principal component analysis (PCA) (28, 29), which maximizes the variance of each component projection, thereby mapping the original input data to an orthogonal coordinate system.

Feature selection aims to select a small part of the input features without losing the information contained in the original features. Our commonly used feature selection methods include: filter, wrapper and embedded methods.

Filter methods are usually classified according to general features, such as looking at the correlation between individual features or independence and output response. For the prediction of drug sensitivity, our commonly used filtering feature selection methods include: (1) The correlation coefficients between genomic features and output responses (30, 52); (2) ReliefF (31, 32) is general and successful attribute estimators. They are able to detect conditional dependencies between attributes, and provide a unified view of attribute estimation in regression and classification. They have the advantages of low computation cost, robust model and noise tolerant, but cannot distinguish redundant features; and (3) Minimum redundancy maximum relevance (mRMR) (33–35), which reduces the redundancy between features and considers a high degree of statistical dependence and output the response. The advantage of filter methods lies in the low computational cost, which usually leads to the problem of bias, which makes it impossible to determine the multivariate feature relationship.

The quality of the selected features in the wrapper methods is affected by the prediction accuracy of the learning algorithm. The wrapper methods usually use high model accuracy to capture features, but the disadvantage of wrapper methods is that they overfit the data. Some commonly used wrapper feature selection methods in drug sensitivity prediction include: (1) Sequential floating forward search (SFFS) (36, 37), where in the forward iteration process, the most representative one will select features from the remaining features. If the removed feature has an impact on the improvement of the objective function, it is provided in the floating part; and (2) Recursive feature elimination (38), which is applicable to all feature models, first sorts the features and eliminates the last feature in turn.

The embedded methods select relevant features through the specific structure of the model, which requires the learning process and feature selection to be interrelated. we usually use embedded methods include: Regularization, which penalizes the norm of feature weights, such as ridge regression (39, 40) penalizing the L-2 norm, LASSO (41, 42, 53) penalize the L-1 norm, and elastic network regularization (43) penalizes the mixture of L-1/2 norm.

In practice, A hybrid methods that combines the most optimal properties of filters and wrappers is usually used. First, the dimension of feature space is reduced by filter methods, and multiple feature subsets can be obtained (44). Then, a wrapper is used to select the optimal feature subset. Several better feature selection methods have been proposed, such as: feature selection based on fuzzy random forest (45), hybrid genetic algorithms (46), hybrid ant colony optimization (47), or hybrid gravity search algorithms (48).

When using hybrid methods, prior knowledge of biological is usually included in the feature section in the process of predicting drug sensitivity. An example is path-based elastic net regularization (49), which incorporates path knowledge in data-driven feature selection. Feature selection based on biological pathways can select the most important features with minimally redundancy, and combine gene expression data with signaling and regulatory pathways (50) or use the activation state of signaling pathways as features (51).

## Matrix Decomposition-Based Methods

Previously molecular synthesis experiments for drug targets were expensive and time-consuming. Therefore, research on drug repositioning requires effective calculation methods, which have proven to be a viable strategy in the field of *in silico* drug discovery. The basic requirement of calculating drug repositioning is to accurately predict the drug and target (DTIs) interaction. Therefore, researchers have proposed some potential methods for predicting DTI in recent years (**Table 2**).

**Table 2:** Matrix decomposition-based methods.

Study	Logistic matrix factorization	Bayesian matrix factorization	Probability matrix factorization	Other	Description	Ref
Liu et al.	**√**				Neighborhood regularized logic matrix factorization (NRLMF)	(54)
Hao et al.	**√**				Dual network integrated logistic matrix factorization	(55)
Ban et al.	**√**				The Hyperparameter Optimization of Improved Neighborhood Regularization Logic Matrix Factorization	(56)
Bolgár et al.		**√**			An extended Bayesian matrix factorization	(57)
Bolgár et al.		**√**			Variational Bayesian multiple kernel logistic matrix factorization (VB-MK-LMF)	(58)
Gonen		**√**			A novel Bayesian formula combing matrix factorization and dimensionality reduction	(59)
Peska et al.		**√**			Matrix decomposition based on Bayesian personalized ranking (BPR)	(60)
Cobanoglu et al.			**√**		Analyze large-scale interactive networks through probability matrix factorization (PMF)	(61)
Cobanoglu et al.			**√**		Online tool based on probability matrix factorization method and DrugBank v3	(62)
Zheng et al.				**√**	Multiple similarities collaborative matrix factorization (MSCMF)	(63)
Wang et al.				**√**	An improved method of disallow the regular term of the drug pathways	(64)
Ezzat et al.				**√**	Two matrix factorization methods using graph regularization	(65)
Peng et al.				**√**	A framework model integrating non-negative matrix factorization, low-rank representation, neighbor interaction profile and sparse representation classification	(34)
Dai et al.				**√**	A matrix factorization model that combines drugs, diseases and genes with feature vectors of the same dimension	(66)

We usually use binary labeling matrix Y to represent drug-target interactions (**Figure 1**). If the drug and the target are in an interaction relationship, it is represented by element 1; If it is not an interactive relationship, it is represented by 0. The difficulty of predicting DTI lies in whether the known elements in y can accurately predict the labels of unknown elements. To solve these problems, assuming similar drugs tend to similar targets, the similarity between drugs and targets can be used to predict DTI, and vice versa.

Liu et al. proposed a neighborhood regularized logic matrix factorization (NRLMF) method (54). This method uses logical matrix decomposition to simulate the interaction probability of each drug target. We further improve the prediction accuracy by neighborhood regularization. The NRLMF model is the most advanced algorithm and has achieved good results on the basis of five 10-fold cross-validation tests. However, The NRLMF model also has some shortcomings, that is, the drug target interaction information is not considered when the model is established. In response to the above problems, Hao et al. proposed a dual-network integrated logic matrix factorization (DNILMF) (55) and integrated drug target profile information into the model. Based on the NRLMF model, Ban et al. used Gaussian process mutual information to accelerate model parameter search (56). Compared with the previous grid search methods, the method based on Gaussian process mutual information saves about 8.94 times of calculation time. When the area under the curve (AUC) is used for evaluation, the prediction accuracy of the two methods is almost the same.

Bolgár et al. proposed an extended Bayesian matrix factorization method (57), which was combined with a new missing not at random (MNAR) data sub-model. Bolgár et al. later proposed variational Bayesian multiple kernel logistic matrix factorization (VB-MK-LMF) (58), which combines multiple kernel learning, weighted observations and graph Laplacian regularization, and it has explicit modeling probability advantage. Gonen proposed a new Bayesian formula that combines matrix factorization and dimensionality reduction (59). This method uses the chemical similarity of drug components and the genomic similarity of target proteins to predict DTI network. Based on Bayesian personalized ranking (BPR) matrix factorization, Peska et al. proposed a method to predict DTIs (60). They extended BPR by including target deviations, developed a technique for analyzing new drugs, and adjusted the content to take into account the structural similarity between the drug and the target.

Cobanoglu et al. used probabilistic matrix factorization (PMF) to analyze large interaction networks (61). They clustered DrugBank drugs based on PMF latent variables. Cobanoglu et al. later built an online tool for evaluating DTIs (62). They use the PMF method and DrugBank v3, and use the GraphLab collaborative filtering toolkit to train potential variable models.

Zheng et al. proposed a method of multiple similarities collaborative matrix factorization (MSCMF) (63). This method allows the collaborative prediction of DTIs through two low-rank matrices and detects similarities that are important for predicting DTIs. Wang et al. proposed a method to replace the regular term of the drug pathway association matrix (L1 norm) with L2-1 norm (64). Compared with the previous iPad method, this method solves the problem of excessively scattered sparsity, and can obtain more optimized performance by identifying effective drug pathway associations.

Ezzat et al. proposed two matrix factorization methods that use graph regularization and consist of two steps (65). First, convert the binary value in the drug-target matrix Y into an interaction likelihood value. Then use matrix factorization to predict DTI. In cross-validation, it is found that the performance of this method is better than the other three other state-of-the-art methods in most cases. They found that their method reasonably predicted missed interactions with “new drugs” and “new target” simulated cases.

Peng et al. proposed a unified model framework (34), which integrates non-negative matrix factorization, low-rank representation, neighbor interaction profile and sparse representation classification. Dai et al. proposed a matrix factorization model (66), which integrates drugs, diseases and genes with feature vectors of the same dimension. Experiments showed that the integration of genomic space is indeed effective.

## Network-Based Methods

In the past decade, network-based approaches (**Figure 1**) have been commonly used to predict drug sensitivity (1, 67). We have summarized some network-based methods in **Table 3**. Due to the increase in drug development costs and the decrease in the number of newly approved drugs, it is necessary to determine the new value of existing drugs. Some network-based methods help design unique drug target combinations and combined drugs therapies (68), and improve the treatment of specific patients through powerful channels (69).

**Table 3:** Network-based methods.

Study	Description	Ref
Huang et al.	A new system calculation tool called DrugComboRanker prioritizes synergistic drug combinations and reveals its mechanism of action	(68)
Dorel et al.	Drug sensitivity prediction based on high-throughput sequencing data and signal network	(69)
Kanehisa M.	KEGG Mapper tool introduction	(70)
Chen et al.	Alternative techniques and tools for analyzing biomolecular networks	(71)
Zhang A.	Discuss current research problems and solutions in protein-protein interaction networks	(72)
Sun P.G.	A multi-level network model integrating drugs, diseases and genes for disease diagnosis, treatment and drug discovery	(73)
Leiserson et al.	A novel algorithm to find mutated subnetworks (HotNet2) is used	(74)
Guney et al.	A metric for quantifying interactions between drugs, targets, and diseases	(84)
Kotlyar et al.	Use networks to characterize genes that are differentially regulated by drugs and find the differences between the genes regulated by drugs and drug targets	(75)
Cheng et al.	A inference method based on topological similarity of drug target bipartite network	(76)
Chen et al.	A network method based on restart random walk	(77)
Chen et al.	A method based on basic network topology measure is used to predict the direct association between drugs and diseases	(78)
Zhou et al.	A weighting method is used that can be directly applied in extracting hidden network information	(79)
Zhou et al.	Hybrid algorithm based on heat-spreading	(80)
Wang et al.	A computing framework based on heterogeneous network model	(81)
Chen et al.	A principled method to improve the prediction performance of two tasks	(82)
Yue et al.	Reorientation of PD drugs with systemic pharmacology framework	(83)

Some researchers have proposed that the relationship between drug application, disease treatment, and genes should be studied (70). Some studies analyzed disease diagnosis, treatment, and drug discovery from the perspective of biological systems and network structure frameworks (71–73). With the development of high-throughput sequencing technology, it is possible to reconstruct cell network and biomolecules. From the cellular level, the reconstructed network will become a hierarchical structure (74). Guney et al. introduced a drug-disease proximity measure that quantifies the interaction between disease and drug targets (84).

Additionally, network-based proximity can help us determine the therapeutic effects of drugs and predict novel drug-disease associations. Kotlyar et al. summarized how drugs disrupt the network, and previous network-based drug effects characterizations included direct binding to partners (75). Drugs can also affect the transcriptome of cells, and networks have been used for the first time to characterize genes differentially regulated by drugs. Cheng et al. constructed a bipartite graph based on the network inference method to predict the interaction between drug and target (76). Chen et al. constructed a general heterogeneous network (77), which was composed of drug and protein, and considered drug-drug chemical similarity, protein-protein sequence similarity and drug-target interaction (78).

The mining potential of drug-disease associations has been consistently used to accelerate the drug repositioning by pharmaceutical companies. Cheng proposed an inference method based on drug-target bipartite network (76), which can be used to predict new targets of known drugs, and described the importance of developing computational methods for predicting potential DTIs. Then, Chen proposed two inference methods, ProbS and HeatS (78), which can predict drug-disease interactions based on the measurement of basic network topology. Methods probs and heats are two methods based on recommendation techniques (79, 80). In order to find the correlation between known drugs and diseases, they solve the above problem by mining the data of drug-disease bipartite network properties. Then, Wang proposed a heterogeneous network model (81). This method uses existing omics data to relocate drugs, diseases and drug targets. This three-layered heterogeneous network model for drug repositioning captured the interrelationships among diseases, drugs, and targets, with the purpose of novel drug usage prediction. Chen et al. provided a principled method to transfer knowledge from these two domains and improve prediction performance for these two tasks (82), With the help of the relationship between drug target disease, this method urges us to consider drug relocation and drug target prediction in drug discovery.

Some researchers have attempted to reposition drugs by targeting network modules through some unique cases, such as a Parkinson’s disease case study. Yue constructed a framework of targeted therapy (83), which combines genome-wide association data with gene co-expression modules of PD disease tissues representing brain regions, and aims to study dysfunctional pathways or processes.

## Reverse Transcriptome Change-Based Methods

Reverse transcriptome change-based methods (**Figure 1**) are methods based on the gene expression profiles induced by drugs. These methods consider the relationship between drugs, genes, and disease. The publicly accessible gene expression profiles currently include Connectivity Map (CMap, http://www.broadinstitute.org/cmap), National Cancer Institute 60 human tumor cell line anticancer drug discovery project (NCI-60 http://dtp.nci.nih.gov/), Library of Integrated Cellular Signatures (LINCS http://www.lincsproject.org/), and Cancer Cell Line Encyclopedia (CCLE http://www.broadinstitute.org/ccle) (2, 52).

It is helpful to facilitate repositioning drugs and chemical compounds with relevant databases. Examples are Gene Expression Omnibus (GEO, http://www.ncbi.nlm.nih.gov/geo/), The Cancer Genome Atlas (TCGA, http://tcga-data.nci.nih.gov/tcga/tcgaHome2.jsp), Gene Expression Database (GXD, http://www.informatics.jax.org/expression.shtml), ArrayExpress (http://www.ebi.ac.uk/arrayexpress), et al. The huge amount of publicly available transcriptome data is enabling the repositioning of drugs and chemical compounds based on the gene expression profiles. We summarize the articles based on the above database in **Table 4**.

**Table 4:** Reverse transcriptome change–based methods.

Study	CMap	NCI-60	LINCS	CCLE	Description	Ref
Lamb et al.	**√**				Establish CMap database	(85)
Iskar et al.	**√**				Developed a pipeline for strict filtering and state-of-the-art normalization for gene expression in CMap	(86)
Hieronymus et al.	**√**				Regulators for predicting cancer phenotypes based on chemical genomics	(87)
Gheeya et al.	**√**				Prediction of the mechanism of action of unknown drugs based on CMap database	(88)
Fayad et al.	**√**				Analysis of MCF-7 gene expression in breast cancer cells based on CMap database	(89)
Wen et al.	**√**				Detection of gene expression changes caused by traditional Chinese medicine ingredients based on CMAP database	(90)
Zhang et al.	**√**				Prediction of molecular mechanism of VPA against CML based on CMAP database	(91)
Brown et al.	**√**				A standard database for drug repositioning (repoDB)	(92)
Shoemaker		**√**			Review the development and use of the NCI-60	(93)
Zaharevitz et al.		**√**			Explain and demonstrate an example of using COMPARE on the web page	(94)
Cheng et al.		**√**			Use the NCI-60 data set to identify new targets for drugs and bioactive compounds on a larger scale	(95)
Nishizuka et al.		**√**			Proteomic profiling of the NCI-60 cancer cell lines using new high-density reverse-phase lysate microarrays	(96)
Subramanian et al.			**√**		Designed a method for cheap and large-scale gene expression analysis (L1000 assay)	(97)
Niepel et al.			**√**		Developed a method for cell growth and survival measurements	(98)
Chen et al.			**√**		Predicted four highly effective compounds capable of reversing liver cancer gene expression, and confirmed that all four compounds are effective against five liver cancer cell lines	(99)
Fallahi-Sichani et al.			**√**		Multi-parameter methods involving analysis	(100)
Barretina et al.				**√**	Created a research tool for predicting genetic variation in cancer drug sensitivity	(27)

Lamb et al. established CMAP database (85), which contains more than 6100 gene expression profiles induced by more than 1300 compounds in four cell lines. The main working idea is to enter a query in the CMap database, using the genome of the drug as a reference. Drug candidates with a positive correlation score (the highest is close to 1) may be considered to be related to the reference drug between downstream regulatory and clinical drug response, while drug candidates with a negative correlation score (the lowest is close to -1) may eventually be considered It is considered that there is no potential correlation or antagonism with the reference drug.

Based on the correlation between drugs and genetic characteristics, we can discover some new drugs indications, and assume that drugs with similar characteristics may have similar therapeutic effects (85). Iskar et al. developed a strict filtering and state-of-the-art normalization pipeline for CMap gene expression (86), and it significantly overcomes cross-batch non-biological experimental variation. Hieronymus et al. proposed a chemical genomic method based on gene expression analysis (87), which can be used to discover and predict compounds with cancer phenotypes, for example, for compounds with gedunin and celastrol activity HSP90 inhibitors are classified. Epoxy anthraquinone derivatives have been found to be a novel DNA topoisomerase inhibitor for the treatment of neuroblastoma and other cancers (88). The alkaloid thaspine from the croton cortex has been shown to play a role in the overexpression of drug efflux transporters in cells, and induce apoptosis of multicellular spheroids cells. It can be used as a dual topoisomerase inhibitor (89).

The molecular mechanism of the traditional Chinese medicinal formula Si-Wu-Tang was discovered through connection maps and gene expression microarray (90). Studies have found that SWT, as an activator and phytoestrogen of Nrf2, it can be used as a non-toxic chemopreventive agent, Through CMap mining and microarray gene expression profiling, the new mechanism of action of traditional Chinese medicine can be verified and discovered. K562 cells exposed to sodium valproate were verified by CMAP database, and it was found that valproate acid could provide certain therapeutic potential in the treatment of leukemia (91). As a combination of approved drugs and failed drugs, repoDB database(http://apps.chiragjpgroup.org/repoDB/) provides researchers with a simplified hypothesis to prove that all novel predictions are false (92).

In the past, anticancer drugs were screened by transplantable animal tumors. In the late 1980s, NCI-60 cell line dataset was developed by the US National Cancer Institute (NCI), aiming at drug discovery *in vitro* (93). The NCI-60 data set involves nine human cancers with a total of 60 cell lines, including: ovarian cancer, prostate cancer, lung cancer, leukemia, colon cancer, breast cancer, etc. The US National Cancer Institute proposed a comparative algorithm to find new compounds with similar mechanisms, or possible mechanisms of action of related compounds (94). The similarity search method of bioactivity map can calculate the similarity between drugs according to the bioactivity map of drugs, and relocate the known drugs according to the similarity (95).

Reverse-phase protein lysate microarray is a method for accurately measuring protein expression levels in NCI-60 cell line. This method has a large number of spots and aims to find a type of molecular with high protein/mRNA correlation (96). In February 2016, NCI-60 was no longer supported because NCI decided to use a patient-derived xenograft (PDX) model instead. Since then, some research institutions and drug companies have begun to build their own model PDX library. EurOPDX composed by 16 European institutions jointly consists of 1500 PDX models, The Jackson Laboratory has 450 PDX models, and the drug screening tool released by Novartis uses 1000 PDX models.

The Library of Integrated Network-based Cellular Signatures (LINCS) program was developed by the US National Institutes of Health to increase understanding of normal and diseased cellular states and how to alter them. Researchers at the LINCS transcription center have released a new version of Connectivity Map, which involves 42000 human cells and more than 1.3 million gene expression profiles. This data set is based on L1000 analysis and aims to reduce the cost of gene expression analysis (97).

In order to analyze the effects of different small molecule drugs on six different breast cancer cell lines, the researchers proposed a method to obtain survival measurements and cell growth. Studies have shown that the survival and growth of certain types of breast cancer cells are affected by drugs, and the existence of differences helps to understand the response of breast cancer patients during treatment (98). Studies have shown that the effects of drugs that can reverse the expression of cancer-related genes are beneficial to the treatment of some cancer models (etc. breast, liver, and colon cancer.) (99). They concluded that the four compounds showed high enough potency to reverse gene expression in liver cancer, and used a system-based method to confirm that the four compounds were effective against the discovered liver cancer cell lines.

It is found that the information obtained by different measurement methods under different drug doses has corresponding uniqueness (100), which is conducive to further exploration of drug effects. When researchers examine the variability of drug effects, they need to consider many factors to expand the way they think about drug activity. The conclusion shows that in the comparison of drug reactions, in addition to the drug effect and price, many factors should be considered, such as clinical concentration near and above the *IC_50_*.

The Cancer Cell Line Encyclopedia (CCLE) project is an effort to conduct a detailed genetic characterization of a large panel of human cancer cell lines (27). CCLE provides public access analysis and visualization of DNA copy number, mRNA expression, mutation data, and other items for approximately 1000 cancer cell lines, as well as the pharmacological profiles of 24 anti-cancer drugs in 50% of cell lines. Barretina et al. developed the research tools for predicting the genetic variation of cancer drug sensitivity and evaluated their systematic analysis methods. They also applied the prediction model method to the cancer genetic subsets that challenge the current treatment methods.

## Discussions

We reviewed the four popular *in silico* methods for drug repositioning based on feature, matrix factorization, network, and reverse transcriptome change. Through the analysis of the four methods, we found that each method has its advantages and limitations and more optimal performance can usually be obtained by combining different methods and strategies.

Despite the creation of some excellent drug repositioning models and methods, the development of robust and satisfactory models is still an indispensable process. One of the main problems is the difficulty in developing functional theoretical models or methods, which is challenging because the construction of such models or methods to simulate biological behavior will have a certain degree of complexity. Due to changes in the conditions and environments that exist during different experiments, the gene expression profile may be difficult to define, which results in data discrepancies in gene expression characteristics. In addition, when genes are used as drug targets, gene expression is not always significant, resulting in inaccurate data. Because of these problems, it is difficult for models or methods to identify potential drug target interactions when following chemical structures or molecular mechanisms.

Another major problem associated with the drug repositioning model is the lack of reliable gold standard datasets. In the process of model building, one scheme is to combine the divided training, validation, and test set with k-fold cross validation and then use the popular evaluation index to evaluate the performance. Another scheme is to establish unique gold standard datasets and then use the evaluation indicators to evaluate the model or method proposed to finally avoid the occurrence of over-fitted problems.

Although there are many challenges in the research of drug repositioning, the integration of multi-source information related to drugs and their side effects, interactions of drugs and diseases, and interactions of drugs and drugs is essential to improve the performance of the drug repositioning domain model. There is still a lack of treatment plans corresponding to the large number of existing diseases, which has inspired more scientific researchers and medical workers to carry out research.

## Author Contributions

XX and PB conceived the concept of the work. BH, FH, CR and PB performed the experiments. BH, FH, and CR wrote the paper. All authors contributed to the article and approved the submitted version.

## Funding

This research was funded by the Natural Science Foundation of China (No. 61803151), the Project of Scientific Research Fund of Hunan Provincial Education Department (Nos. 19A060 and 19C0185), and the Hunan Provincial Innovation Platform and Talents Program (No. 2018RS3105).

## Conflict of Interest

CR was employed by Geneis Beijing Co., Ltd.

The remaining authors declare that the research was conducted in the absence of any commercial or financial relationships that could be construed as a potential conflict of interest.
